# {4,4′-Dimeth­oxy-2,2′-[1,1′-(ethane-1,2-diyldinitrilo)diethyl­idyne]diphenolato}nickel(II) hemihydrate

**DOI:** 10.1107/S1600536808023362

**Published:** 2008-07-31

**Authors:** Hoong-Kun Fun, Reza Kia

**Affiliations:** a X-ray Crystallography Unit, School of Physics, Universiti Sains Malaysia, 11800 USM, Penang, Malaysia

## Abstract

In the title complex, [Ni(C_20_H_22_N_2_O_4_)]·0.5H_2_O, the Ni^II^ ion is in a slightly distorted square-planar geometry involving an N_2_O_2_ atom set of the tetra­dentate Schiff base ligand. The asymmetric unit contains one mol­ecule of the complex and half a water solvent mol­ecule. The solvent water mol­ecule lies on a crystallographic twofold rotation axis. An inter­molecular O—H⋯O hydrogen bond forms an *R*_2_^1^(4) ring motif involving a bifurcated hydrogen bond to the phenolate O atoms of the complex. In the crystal structure, mol­ecules are linked by π–π stacking inter­actions, with centroid–centroid distances in the range 3.5310 (11)–3.7905 (12) Å, forming extended chains along the *b* axis. In addition, there are Ni⋯Ni and Ni⋯N inter­actions [3.4404 (4)–4.1588 (4) and 3.383 (2)–3.756 (2) Å, respectively] which are shorter than the sum of the van der Waals radii of the relevant atoms. Further stabilization of the crystal structure is attained by weak inter­molecular C—H⋯O and C—H⋯π inter­actions.

## Related literature

For bond-length data, see: Allen *et al.* (1987 ▶). For hydrogen-bond motifs, see: Bernstein *et al.* (1995 ▶). For related structures see, for example: Clark *et al.* (1968 ▶, 1969 ▶, 1970 ▶); Hodgson (1975 ▶). For applications and bioactivities see, for example: Elmali *et al.* (2000 ▶); Blower (1998 ▶); Granovski *et al.* (1993 ▶); Li & Chang (1991 ▶); Shahrokhian *et al.* (2000 ▶); Fun & Kia (2008 ▶).
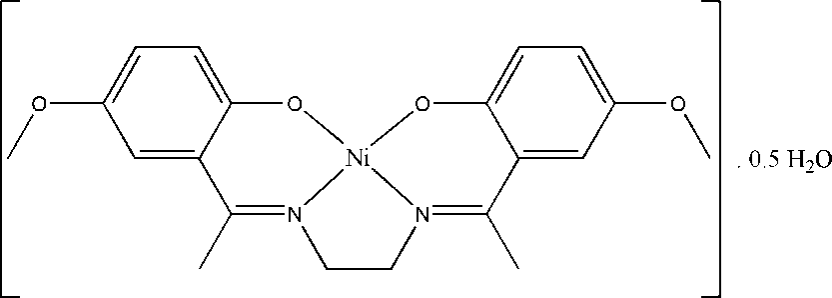


## Experimental

### 

#### Crystal data

[Ni(C_20_H_22_N_2_O_4_)]·0.5H_2_O
*M* *_r_* = 422.11
Monoclinic, 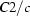

*a* = 29.1721 (7) Å
*b* = 7.3032 (2) Å
*c* = 17.2833 (4) Å
β = 101.323 (1)°
*V* = 3610.53 (16) Å^3^
*Z* = 8
Mo *K*α radiation
μ = 1.11 mm^−1^
*T* = 100.0 (1) K
0.33 × 0.18 × 0.15 mm

#### Data collection

Bruker SMART APEXII CCD area-detector diffractometer
Absorption correction: multi-scan (*SADABS*; Bruker, 2005 ▶) *T*_min_ = 0.712, *T*_max_ = 0.853
21087 measured reflections
5319 independent reflections
4166 reflections with *I* > 2˘*I*)
*R*_int_ = 0.042

#### Refinement

*R*[*F* ^2^ > 2σ(*F* ^2^)] = 0.051
*wR*(*F* ^2^) = 0.138
*S* = 1.04
5319 reflections
253 parameters
H-atom parameters constrained
Δρ_max_ = 1.43 e Å^−3^
Δρ_min_ = −0.90 e Å^−3^

### 

Data collection: *APEX2* (Bruker, 2005 ▶); cell refinement: *APEX2*; data reduction: *SAINT* (Bruker, 2005 ▶); program(s) used to solve structure: *SHELXTL* (Sheldrick, 2008 ▶); program(s) used to refine structure: *SHELXTL*; molecular graphics: *SHELXTL*; software used to prepare material for publication: *SHELXTL* and *PLATON* (Spek, 2003 ▶).

## Supplementary Material

Crystal structure: contains datablocks global, I. DOI: 10.1107/S1600536808023362/lh2663sup1.cif

Structure factors: contains datablocks I. DOI: 10.1107/S1600536808023362/lh2663Isup2.hkl

Additional supplementary materials:  crystallographic information; 3D view; checkCIF report

## Figures and Tables

**Table:** 

Ni1—O2	1.8201 (16)
Ni1—O1	1.8315 (15)
Ni1—N1	1.8575 (19)
Ni1—N2	1.8617 (19)

**Table:** 

Ni1⋯Ni1^i^	3.4404 (4)
Ni1⋯Ni1^ii^	4.1588 (4)
Ni1⋯N1^i^	3.383 (2)
Ni1⋯N2^i^	3.756 (2)
Ni1⋯N2^ii^	3.728 (2)
*Cg*1⋯*Cg*3^iii^	3.7905 (12)
*Cg*3⋯*Cg*4^iv^	3.5310 (11)
*Cg*4⋯*Cg*4^iii^	3.6152 (11)

**Table:** 

O2—Ni1—O1	81.89 (7)
O2—Ni1—N1	175.30 (8)
O1—Ni1—N1	94.47 (8)
O2—Ni1—N2	93.96 (8)
O1—Ni1—N2	175.13 (8)
N1—Ni1—N2	89.81 (8)

Symmetry codes: (i) 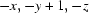; (ii) 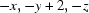; (iii) 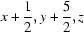; (iv) 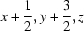. *Cg*1, *Cg*3, and *Cg*4 are the centroids of the C11–C16, Ni1-O1-C1-C6-C7-N1 and Ni1-O2-C16-C11-C10-N2 rings, respectively.

**Table 2:** Hydrogen-bond geometry (Å, °)

*D*—H⋯*A*	*D*—H	H⋯*A*	*D*⋯*A*	*D*—H⋯*A*
O1*W*—H1*W*1⋯O1	0.84	2.41	3.1173 (19)	143
O1*W*—H1*W*1⋯O2	0.84	2.21	2.9077 (16)	141
C8—H8*A*⋯O2^i^	0.97	2.47	3.319 (3)	146
C9—H9*A*⋯O1*W*^ii^	0.97	2.52	3.407 (3)	152
C18—H18*B*⋯*Cg*1^iv^	0.96	2.71	3.385 (2)	127
C19—H19*C*⋯*Cg*2^iv^	0.96	2.81	3.652 (3)	146

Symmetry codes: (i) 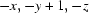; (ii) 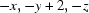; (iv) 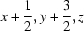. *Cg*1 and *Cg*2 are centroids of the C11–C16 and C1–C6 benzene rings, respectively.

**Fig. 1.**
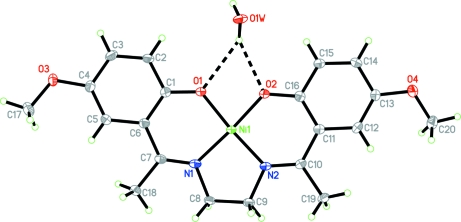
The molecular structure of the title compound, showing 50% probability displacement ellipsoids and the atomic numbering. Intermolecular hydrogen bonds are drawn as dashed lines.

**Fig. 2.**
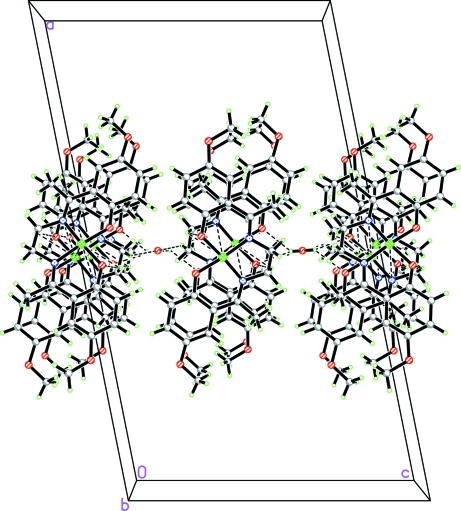
The crystal packing of (I), viewd down the b axis, showing stacking of molecules along the b axis. Intramolecular and intermolecular interactions are drawn as dashed lines.

## supplementary crystallographic information

### Comment

Schiff base complexes are some of the most important stereochemical models in transition metal coordination chemistry, with their ease of preparation and structural variations (Granovski *et al.*, 1993). Transition metal complexes of Schiff base ligands are always of interest since they exhibit a marked tendency to oligomerize, thus leading to novel structural types, and also display a wide variety of magnetic properties. Many of the reported structural investigations of these complexes are discussed in some details in a review (Hodgson, 1975). Metal derivatives of Schiff bases have been studied extensively, and Cu(II) and Ni(II) complexes play a major role in both synthetic and structural research (Elmali *et al.*, 2000; Blower, 1993; Fun & Kia, 2008; Granovski *et al.*, 1993; Li & Chang, 1991; Shahrokhian *et al.*, 2000). Tetradentate Schiff base metal complexes may form *trans* or *cis* planar or tetrahedral structures (Elmali *et al.*, 2000).

In the title compound (I, Fig. 1), the Ni^II^ ion shows a sligthly distorted square-planar geometry which is coordinated by two imine N atoms and two phenol O atoms of the tetradentate Schiff base ligand. An intermolecular O—H···O hydrogen bond forms a four-membered ring, producing a *R*^1^_2_(4) ring motif (Bernstein *et al.*, 1995). The bond lengths are within the normal ranges (Allen *et al.*, 1987). The asymmetric unit of the compound contains one molecule of the complex, and one-half of the water solvent. The latter shows bifurcated hydrogen bond which is connected to the phenolato oxygen atoms of the complex. Atoms C8 and C9 are significantly out of the plane, as indicated by the torsion angle N1–C8–C9–N2, which is -23.6 (3)°. The dihedral angle betwen two benzene rings is 5.13 (11)°. In the crystal structure, (Fig. 2), the molecules are form 1-D extended chains along the *b* axis with Ni···Ni and Ni···N separations (Table 2) of 3.4404 (4) – 4.1588 (4), and 3.383 (2) – 3.756 (2) Å, and short intermolecular distances between the centroids of the six-membered rings [3.5310 (11) – 3.7905 (12) Å], respectively. The Ni···Ni dimeric separations are significantly shorter than the sum of the van der Waals radii of two Ni atoms (4.60 Å). The crystal packing is stabilized by intermolecular O—H···O (*x* 2), and C—H···O (*x* 2) hydrogen bonds, and weak intermolecular C—H···π interactions.

### Experimental

A chloroform solution (40 ml) of the ligand (1 mmol, 354 mg) was added to a methanol solution (20 ml) of NiCl_2_.6H_2_O (1.05 mmol, 237 mg). The mixture was refluxed for 30 min and then filtered. After keeping the filtrate in air for 4 d, red block-shaped crystals were formed at the bottom of the vessel on slow evaporation of the solvent.

### Refinement

The water H-atoms are located from the difference Fourier map and refined as riding with the parent atom with an isotropic thermal parameter 1.5 times that of the parent atom. The rest of the hydrogen atoms were positioned geometrically [C—H = 0.93–97 Å] and refined using a riding model. A rotating-group model was used for the methyl groups.

### Crystal data

**Table:**

[Ni(C_20_H_22_N_2_O_4_)]·0.5H_2_O	*F*_000_ = 1776
*M**_r_* = 422.11	*D*_x_ = 1.557 Mg m^−^^3^
Monoclinic, *C*2/*c*	Mo *K*α radiation λ = 0.71073 Å
Hall symbol: -C 2yc	Cell parameters from 6474 reflections
*a* = 29.1721 (7) Å	θ = 2.4–28.5º
*b* = 7.3032 (2) Å	µ = 1.11 mm^−^^1^
*c* = 17.2833 (4) Å	*T* = 100.0 (1) K
β = 101.323 (1)º	Block, red
*V* = 3610.53 (16) Å^3^	0.33 × 0.18 × 0.15 mm
*Z* = 8	

### Data collection

**Table:**

Bruker SMART APEXII CCD area-detector diffractometer	5319 independent reflections
Radiation source: fine-focus sealed tube	4166 reflections with *I* > 2˘*I*)
Monochromator: graphite	*R*_int_ = 0.042
*T* = 100.0(1) K	θ_max_ = 30.2º
φ and ω scans	θ_min_ = 1.4º
Absorption correction: multi-scan(SADABS; Bruker, 2005)	*h* = −35→41
*T*_min_ = 0.712, *T*_max_ = 0.853	*k* = −10→8
21087 measured reflections	*l* = −24→24

### Refinement

**Table:**

Refinement on *F*^2^	Secondary atom site location: difference Fourier map
Least-squares matrix: full	Hydrogen site location: inferred from neighbouring sites
*R*[*F*^2^ > 2σ(*F*^2^)] = 0.051	H-atom parameters constrained
*wR*(*F*^2^) = 0.138	*w* = 1/[σ^2^(*F*_o_^2^) + (0.0817*P*)^2^ + 1.6212*P*] where *P* = (*F*_o_^2^ + 2*F*_c_^2^)/3
*S* = 1.04	(Δ/σ)_max_ < 0.001
5319 reflections	Δρ_max_ = 1.43 e Å^−^^3^
253 parameters	Δρ_min_ = −0.90 e Å^−^^3^
Primary atom site location: structure-invariant direct methods	Extinction correction: none

### Special details

**Table:**

Experimental. The low-temperature data was collected with the Oxford Cyrosystem Cobra low-temperature attachment.
Geometry. All e.s.d.'s (except the e.s.d. in the dihedral angle between two l.s. planes) are estimated using the full covariance matrix. The cell e.s.d.'s are taken into account individually in the estimation of e.s.d.'s in distances, angles and torsion angles; correlations between e.s.d.'s in cell parameters are only used when they are defined by crystal symmetry. An approximate (isotropic) treatment of cell e.s.d.'s is used for estimating e.s.d.'s involving l.s. planes.
Refinement. Refinement of *F*^2^ against ALL reflections. The weighted *R*-factor *wR* and goodness of fit *S* are based on *F*^2^, conventional *R*-factors *R* are based on *F*, with *F* set to zero for negative *F*^2^. The threshold expression of *F*^2^ > σ(*F*^2^) is used only for calculating *R*-factors(gt) *etc*. and is not relevant to the choice of reflections for refinement. *R*-factors based on *F*^2^ are statistically about twice as large as those based on *F*, and *R*- factors based on ALL data will be even larger.

### Fractional atomic coordinates and isotropic or equivalent isotropic displacement parameters (Å^2^)

**Table:**

	*x*	*y*	*z*	*U*_iso_*/*U*_eq_	
Ni1	0.013248 (10)	0.72441 (4)	0.025207 (15)	0.01283 (11)	
O1	0.04524 (6)	0.6694 (2)	0.12462 (9)	0.0166 (3)	
O2	−0.02834 (6)	0.8182 (2)	0.08056 (9)	0.0163 (3)	
O3	0.22776 (6)	0.4706 (3)	0.23839 (11)	0.0386 (6)	
O4	−0.20747 (6)	1.0823 (3)	−0.00397 (10)	0.0263 (4)	
N1	0.05957 (7)	0.6362 (3)	−0.02489 (11)	0.0148 (4)	
N2	−0.02394 (7)	0.7776 (3)	−0.07261 (11)	0.0138 (4)	
C1	0.08918 (8)	0.6183 (3)	0.14511 (13)	0.0146 (4)	
C2	0.10797 (8)	0.6091 (3)	0.22710 (13)	0.0174 (5)	
H2A	0.0889	0.6372	0.2627	0.021*	
C3	0.15355 (9)	0.5599 (4)	0.25526 (14)	0.0229 (5)	
H3A	0.1651	0.5554	0.3094	0.027*	
C4	0.18251 (9)	0.5167 (4)	0.20272 (14)	0.0221 (5)	
C5	0.16555 (8)	0.5227 (3)	0.12301 (13)	0.0184 (5)	
H5A	0.1852	0.4928	0.0885	0.022*	
C6	0.11850 (8)	0.5736 (3)	0.09201 (13)	0.0148 (4)	
C7	0.10115 (8)	0.5758 (3)	0.00639 (13)	0.0148 (5)	
C8	0.04336 (8)	0.6242 (3)	−0.11176 (12)	0.0155 (5)	
H8A	0.0355	0.4982	−0.1266	0.019*	
H8B	0.0683	0.6625	−0.1379	0.019*	
C9	0.00088 (9)	0.7449 (3)	−0.13816 (13)	0.0167 (5)	
H9A	0.0106	0.8611	−0.1568	0.020*	
H9B	−0.0202	0.6866	−0.1816	0.020*	
C10	−0.06551 (8)	0.8514 (3)	−0.08899 (13)	0.0147 (4)	
C11	−0.09141 (8)	0.8976 (3)	−0.02736 (13)	0.0150 (4)	
C12	−0.13832 (8)	0.9617 (3)	−0.04730 (13)	0.0165 (5)	
H12A	−0.1531	0.9677	−0.1001	0.020*	
C13	−0.16223 (8)	1.0148 (3)	0.00996 (14)	0.0182 (5)	
C14	−0.14041 (8)	1.0085 (3)	0.08966 (13)	0.0184 (5)	
H14A	−0.1562	1.0488	0.1283	0.022*	
C15	−0.09594 (8)	0.9432 (3)	0.11049 (13)	0.0161 (5)	
H15A	−0.0819	0.9378	0.1636	0.019*	
C16	−0.07050 (8)	0.8834 (3)	0.05342 (13)	0.0152 (5)	
C17	0.26105 (9)	0.4494 (4)	0.19015 (16)	0.0292 (6)	
H17A	0.2906	0.4159	0.2221	0.044*	
H17B	0.2643	0.5626	0.1636	0.044*	
H17C	0.2510	0.3552	0.1519	0.044*	
C18	0.13369 (9)	0.5047 (3)	−0.04465 (14)	0.0185 (5)	
H18A	0.1160	0.4753	−0.0961	0.028*	
H18B	0.1493	0.3968	−0.0210	0.028*	
H18C	0.1565	0.5968	−0.0493	0.028*	
C19	−0.08767 (8)	0.8943 (3)	−0.17351 (13)	0.0187 (5)	
H19A	−0.0638	0.9289	−0.2019	0.028*	
H19B	−0.1095	0.9932	−0.1747	0.028*	
H19C	−0.1038	0.7879	−0.1977	0.028*	
C20	−0.23260 (9)	1.0747 (4)	−0.08316 (15)	0.0263 (6)	
H20A	−0.2635	1.1232	−0.0858	0.039*	
H20B	−0.2347	0.9498	−0.1009	0.039*	
H20C	−0.2166	1.1459	−0.1163	0.039*	
O1W	0.0000	0.8705 (3)	0.2500	0.0218 (5)	
H1W1	−0.0017	0.8066	0.2094	0.033*	

### Atomic displacement parameters (Å^2^)

**Table:**

	*U*^11^	*U*^22^	*U*^33^	*U*^12^	*U*^13^	*U*^23^
Ni1	0.01457 (17)	0.01553 (18)	0.00876 (15)	0.00044 (11)	0.00319 (11)	−0.00026 (10)
O1	0.0164 (8)	0.0229 (9)	0.0106 (7)	0.0030 (7)	0.0031 (6)	−0.0001 (6)
O2	0.0169 (8)	0.0207 (9)	0.0114 (7)	0.0043 (7)	0.0026 (6)	−0.0012 (6)
O3	0.0139 (9)	0.0829 (18)	0.0188 (9)	0.0118 (10)	0.0030 (8)	0.0066 (10)
O4	0.0159 (9)	0.0410 (12)	0.0216 (9)	0.0080 (8)	0.0026 (7)	−0.0027 (8)
N1	0.0197 (10)	0.0146 (10)	0.0110 (8)	−0.0028 (8)	0.0055 (7)	−0.0004 (7)
N2	0.0176 (10)	0.0149 (9)	0.0095 (8)	−0.0008 (8)	0.0042 (7)	−0.0002 (7)
C1	0.0161 (11)	0.0139 (11)	0.0139 (10)	−0.0014 (9)	0.0029 (8)	0.0002 (8)
C2	0.0187 (12)	0.0222 (12)	0.0124 (10)	−0.0009 (9)	0.0058 (9)	−0.0007 (9)
C3	0.0200 (13)	0.0356 (15)	0.0121 (11)	−0.0034 (11)	0.0007 (9)	0.0027 (10)
C4	0.0146 (12)	0.0341 (15)	0.0173 (11)	0.0013 (11)	0.0022 (9)	0.0037 (10)
C5	0.0155 (12)	0.0249 (13)	0.0155 (11)	0.0008 (10)	0.0050 (9)	−0.0003 (9)
C6	0.0158 (11)	0.0154 (11)	0.0134 (10)	−0.0018 (9)	0.0034 (8)	−0.0002 (8)
C7	0.0184 (12)	0.0136 (11)	0.0135 (10)	−0.0024 (9)	0.0055 (9)	−0.0005 (8)
C8	0.0182 (11)	0.0178 (11)	0.0116 (10)	0.0007 (9)	0.0056 (8)	−0.0010 (8)
C9	0.0187 (12)	0.0212 (12)	0.0111 (10)	−0.0013 (9)	0.0050 (9)	0.0002 (8)
C10	0.0170 (11)	0.0141 (11)	0.0124 (10)	−0.0022 (9)	0.0012 (8)	0.0009 (8)
C11	0.0184 (12)	0.0128 (11)	0.0141 (10)	−0.0017 (9)	0.0042 (9)	−0.0004 (8)
C12	0.0165 (12)	0.0169 (12)	0.0145 (10)	−0.0004 (9)	−0.0009 (9)	0.0026 (8)
C13	0.0150 (11)	0.0212 (12)	0.0178 (11)	0.0033 (9)	0.0023 (9)	0.0011 (9)
C14	0.0193 (12)	0.0219 (13)	0.0154 (11)	0.0016 (10)	0.0073 (9)	−0.0011 (9)
C15	0.0186 (12)	0.0171 (11)	0.0126 (10)	−0.0002 (9)	0.0032 (9)	−0.0003 (8)
C16	0.0171 (11)	0.0125 (11)	0.0163 (10)	−0.0001 (9)	0.0039 (9)	−0.0001 (8)
C17	0.0186 (13)	0.0424 (17)	0.0284 (14)	0.0081 (12)	0.0089 (11)	0.0058 (12)
C18	0.0191 (12)	0.0210 (12)	0.0165 (11)	0.0026 (10)	0.0064 (9)	−0.0015 (9)
C19	0.0214 (12)	0.0215 (12)	0.0124 (10)	0.0021 (10)	0.0014 (9)	0.0013 (9)
C20	0.0183 (13)	0.0335 (15)	0.0246 (13)	0.0054 (11)	−0.0019 (10)	−0.0003 (11)
O1W	0.0307 (14)	0.0245 (14)	0.0100 (10)	0.000	0.0039 (10)	0.000

### Geometric parameters (Å, °)

**Table:**

Ni1—O2	1.8201 (16)	C8—H8B	0.9700
Ni1—O1	1.8315 (15)	C9—H9A	0.9700
Ni1—N1	1.8575 (19)	C9—H9B	0.9700
Ni1—N2	1.8617 (19)	C10—C11	1.461 (3)
O1—C1	1.315 (3)	C10—C19	1.510 (3)
O2—C16	1.316 (3)	C11—C16	1.413 (3)
O3—C4	1.384 (3)	C11—C12	1.423 (3)
O3—C17	1.407 (3)	C12—C13	1.374 (3)
O4—C13	1.385 (3)	C12—H12A	0.9300
O4—C20	1.421 (3)	C13—C14	1.400 (3)
N1—C7	1.304 (3)	C14—C15	1.363 (3)
N1—C8	1.486 (3)	C14—H14A	0.9300
N2—C10	1.307 (3)	C15—C16	1.415 (3)
N2—C9	1.479 (3)	C15—H15A	0.9300
C1—C6	1.410 (3)	C17—H17A	0.9600
C1—C2	1.417 (3)	C17—H17B	0.9600
C2—C3	1.371 (3)	C17—H17C	0.9600
C2—H2A	0.9300	C18—H18A	0.9600
C3—C4	1.392 (3)	C18—H18B	0.9600
C3—H3A	0.9300	C18—H18C	0.9600
C4—C5	1.370 (3)	C19—H19A	0.9600
C5—C6	1.422 (3)	C19—H19B	0.9600
C5—H5A	0.9300	C19—H19C	0.9600
C6—C7	1.467 (3)	C20—H20A	0.9600
C7—C18	1.509 (3)	C20—H20B	0.9600
C8—C9	1.516 (3)	C20—H20C	0.9600
C8—H8A	0.9700	O1W—H1W1	0.8359
			
Ni1···Ni1^i^	3.4404 (4)	Ni1···N2^ii^	3.728 (2)
Ni1···Ni1^ii^	4.1588 (4)	Cg1···Cg3^iii^	3.7905 (12)
Ni1···N1^i^	3.383 (2)	Cg3···Cg4^iv^	3.5310 (11)
Ni1···N2^i^	3.756 (2)	Cg4···Cg4^iii^	3.6152 (11)
			
O2—Ni1—O1	81.89 (7)	C8—C9—H9B	109.4
O2—Ni1—N1	175.30 (8)	H9A—C9—H9B	108.0
O1—Ni1—N1	94.47 (8)	N2—C10—C11	121.9 (2)
O2—Ni1—N2	93.96 (8)	N2—C10—C19	119.9 (2)
O1—Ni1—N2	175.13 (8)	C11—C10—C19	118.2 (2)
N1—Ni1—N2	89.81 (8)	C16—C11—C12	118.1 (2)
C1—O1—Ni1	127.23 (14)	C16—C11—C10	121.2 (2)
C16—O2—Ni1	128.42 (14)	C12—C11—C10	120.6 (2)
C4—O3—C17	118.2 (2)	C13—C12—C11	121.2 (2)
C13—O4—C20	116.57 (19)	C13—C12—H12A	119.4
C7—N1—C8	118.98 (19)	C11—C12—H12A	119.4
C7—N1—Ni1	128.81 (16)	C12—C13—O4	125.2 (2)
C8—N1—Ni1	112.03 (14)	C12—C13—C14	120.2 (2)
C10—N2—C9	118.29 (19)	O4—C13—C14	114.6 (2)
C10—N2—Ni1	129.30 (16)	C15—C14—C13	119.8 (2)
C9—N2—Ni1	112.11 (15)	C15—C14—H14A	120.1
O1—C1—C6	125.0 (2)	C13—C14—H14A	120.1
O1—C1—C2	116.6 (2)	C14—C15—C16	121.8 (2)
C6—C1—C2	118.4 (2)	C14—C15—H15A	119.1
C3—C2—C1	121.7 (2)	C16—C15—H15A	119.1
C3—C2—H2A	119.2	O2—C16—C11	124.8 (2)
C1—C2—H2A	119.2	O2—C16—C15	116.4 (2)
C2—C3—C4	119.9 (2)	C11—C16—C15	118.8 (2)
C2—C3—H3A	120.1	O3—C17—H17A	109.5
C4—C3—H3A	120.1	O3—C17—H17B	109.5
C5—C4—O3	125.5 (2)	H17A—C17—H17B	109.5
C5—C4—C3	120.2 (2)	O3—C17—H17C	109.5
O3—C4—C3	114.4 (2)	H17A—C17—H17C	109.5
C4—C5—C6	121.2 (2)	H17B—C17—H17C	109.5
C4—C5—H5A	119.4	C7—C18—H18A	109.5
C6—C5—H5A	119.4	C7—C18—H18B	109.5
C1—C6—C5	118.6 (2)	H18A—C18—H18B	109.5
C1—C6—C7	121.4 (2)	C7—C18—H18C	109.5
C5—C6—C7	119.9 (2)	H18A—C18—H18C	109.5
N1—C7—C6	122.0 (2)	H18B—C18—H18C	109.5
N1—C7—C18	121.0 (2)	C10—C19—H19A	109.5
C6—C7—C18	117.0 (2)	C10—C19—H19B	109.5
N1—C8—C9	110.42 (18)	H19A—C19—H19B	109.5
N1—C8—H8A	109.6	C10—C19—H19C	109.5
C9—C8—H8A	109.6	H19A—C19—H19C	109.5
N1—C8—H8B	109.6	H19B—C19—H19C	109.5
C9—C8—H8B	109.6	O4—C20—H20A	109.5
H8A—C8—H8B	108.1	O4—C20—H20B	109.5
N2—C9—C8	110.95 (18)	H20A—C20—H20B	109.5
N2—C9—H9A	109.4	O4—C20—H20C	109.5
C8—C9—H9A	109.4	H20A—C20—H20C	109.5
N2—C9—H9B	109.4	H20B—C20—H20C	109.5
			
O2—Ni1—O1—C1	−166.3 (2)	C8—N1—C7—C18	3.6 (3)
N1—Ni1—O1—C1	10.7 (2)	Ni1—N1—C7—C18	178.43 (16)
N2—Ni1—O1—C1	162.0 (9)	C1—C6—C7—N1	7.0 (3)
O1—Ni1—O2—C16	−172.8 (2)	C5—C6—C7—N1	−174.1 (2)
N1—Ni1—O2—C16	147.8 (9)	C1—C6—C7—C18	−173.1 (2)
N2—Ni1—O2—C16	4.6 (2)	C5—C6—C7—C18	5.8 (3)
O2—Ni1—N1—C7	33.3 (11)	C7—N1—C8—C9	−164.8 (2)
O1—Ni1—N1—C7	−5.7 (2)	Ni1—N1—C8—C9	19.5 (2)
N2—Ni1—N1—C7	176.6 (2)	C10—N2—C9—C8	−168.1 (2)
O2—Ni1—N1—C8	−151.6 (9)	Ni1—N2—C9—C8	17.5 (2)
O1—Ni1—N1—C8	169.41 (15)	N1—C8—C9—N2	−23.6 (3)
N2—Ni1—N1—C8	−8.25 (15)	C9—N2—C10—C11	−176.6 (2)
O2—Ni1—N2—C10	−1.8 (2)	Ni1—N2—C10—C11	−3.4 (3)
O1—Ni1—N2—C10	29.6 (10)	C9—N2—C10—C19	2.3 (3)
N1—Ni1—N2—C10	−179.0 (2)	Ni1—N2—C10—C19	175.59 (16)
O2—Ni1—N2—C9	171.73 (15)	N2—C10—C11—C16	7.2 (4)
O1—Ni1—N2—C9	−156.8 (9)	C19—C10—C11—C16	−171.8 (2)
N1—Ni1—N2—C9	−5.46 (16)	N2—C10—C11—C12	−174.0 (2)
Ni1—O1—C1—C6	−8.5 (3)	C19—C10—C11—C12	7.0 (3)
Ni1—O1—C1—C2	171.10 (16)	C16—C11—C12—C13	2.3 (3)
O1—C1—C2—C3	−179.1 (2)	C10—C11—C12—C13	−176.5 (2)
C6—C1—C2—C3	0.6 (4)	C11—C12—C13—O4	178.6 (2)
C1—C2—C3—C4	−0.3 (4)	C11—C12—C13—C14	0.7 (4)
C17—O3—C4—C5	8.2 (4)	C20—O4—C13—C12	7.9 (4)
C17—O3—C4—C3	−171.5 (2)	C20—O4—C13—C14	−174.1 (2)
C2—C3—C4—C5	−0.2 (4)	C12—C13—C14—C15	−2.4 (4)
C2—C3—C4—O3	179.5 (2)	O4—C13—C14—C15	179.5 (2)
O3—C4—C5—C6	−179.3 (3)	C13—C14—C15—C16	1.0 (4)
C3—C4—C5—C6	0.4 (4)	Ni1—O2—C16—C11	−2.0 (3)
O1—C1—C6—C5	179.2 (2)	Ni1—O2—C16—C15	178.21 (16)
C2—C1—C6—C5	−0.4 (3)	C12—C11—C16—O2	176.6 (2)
O1—C1—C6—C7	−1.9 (4)	C10—C11—C16—O2	−4.6 (4)
C2—C1—C6—C7	178.5 (2)	C12—C11—C16—C15	−3.7 (3)
C4—C5—C6—C1	−0.1 (4)	C10—C11—C16—C15	175.2 (2)
C4—C5—C6—C7	−179.0 (2)	C14—C15—C16—O2	−178.2 (2)
C8—N1—C7—C6	−176.5 (2)	C14—C15—C16—C11	2.1 (4)
Ni1—N1—C7—C6	−1.6 (3)		

Symmetry codes: (i) −*x*, −*y*+1, −*z*; (ii) −*x*, −*y*+2, −*z*; (iii) *x*+1/2, *y*+5/2, *z*; (iv) *x*+1/2, *y*+3/2, *z*.

### Hydrogen-bond geometry (Å, °)

**Table:**

*D*—H···*A*	*D*—H	H···*A*	*D*···*A*	*D*—H···*A*
O1W—H1W1···O1	0.84	2.41	3.1173 (19)	143
O1W—H1W1···O2	0.84	2.21	2.9077 (16)	141
C8—H8A···O2^i^	0.97	2.47	3.319 (3)	146
C9—H9A···O1W^ii^	0.97	2.52	3.407 (3)	152
C18—H18B···Cg1^iv^	0.96	2.71	3.385 (2)	127
C19—H19C···Cg2^iv^	0.96	2.81	3.652 (3)	146

Symmetry codes: (i) −*x*, −*y*+1, −*z*; (ii) −*x*, −*y*+2, −*z*; (iv) *x*+1/2, *y*+3/2, *z*.
